# Nitrogen migration and transformation characteristics of the soil in karst areas under the combined application of oxalic acid and urea inhibitors

**DOI:** 10.3389/fpls.2024.1386912

**Published:** 2024-05-16

**Authors:** Wang Jiafeng, Cai Qiuliang

**Affiliations:** ^1^ Guangxi Key Laboratory of Biology for Mango, Agriculture and Food Engineering College, Industrial College of Subtropical Characteristic Agriculture, Baise University, Guangxi, Baise, China; ^2^ College of Biological and Food Engineering, Chongqing Three Gorges University, Chongqing, China

**Keywords:** moisture content, nitrogen fertilizer, transformation law, yellow-brown soil, oxalic acid

## Abstract

**Objective:**

We investigated the horizontal migration and transformation of nitrogen in soil with oxalic acid and inhibitors (e.g., nitrification inhibitors, DMPP, urease inhibitors, and NBPT) under different soil water contents to provide a basis for the efficient utilization of nitrogen fertilizer in agricultural production in karst areas.

**Methods:**

Four nitrogen fertilizers (e.g., ammonium bicarbonate, ammonium sulfate, ammonium chloride, and urea) were applied separately and combined with oxalic acid, DMPP, and NBPT. The ammonium and nitrate nitrogen contents in the different soil layers were measured. The soil columns were cultured through an indoor soil column simulation at water content levels of 30%, 40%, and flooded (50%) for 30 days.

**Results:**

Ammonium bicarbonate with inhibitors increased soil NH_4_^+^-N content by 15.42–21.12%. Ammonium sulfate with oxalic acid or NBPT increased soil NH_4_^+^-N content by 27.56–52.25% at 30% and 40% moisture content treatments, compared to ammonium sulfate alone. Urea with DMPP application significantly increased soil NH_4_^+^-N content by 11.93–14.87% at 40% water content and flooded conditions. In all treatments, the NH_4_^+^-N content in the soil treated with 30% water content of ammonium chloride with oxalic acid was the highest. The NH_4_^+^-N content showed a decreasing trend with an increase in the water content. The NO_3_^−^-N content in soil treated with ammonium bicarbonate and DMPP was higher than that treated with other nitrogen fertilizers at 30% moisture. The NO_3_^−^-N content decreased with increased water content. Under all treatments, ammonium chloride with oxalic acid had the highest percentage of soil NH_4_^+^-N and soil soluble inorganic nitrogen at 30% water content, with 55.29% and 55.97%, respectively.

**Conclusion:**

Among the nitrogen fertilizer treatments, the soil NH_4_^+^-N content increased in ammonium bicarbonate with DMPP or NBPT, ammonium sulfate with oxalic acid or NBPT, and urea with DMPP. The four nitrogen fertilizers with DMPP increased the soil NO_3_^−^-N content. Nitrogen fertilizer combined with oxalic acid and inhibitors could effectively improve the effective use of nitrogen fertilizer.

## 1 Introduction

The karst ecosystem is one of the three most fragile ecosystems worldwide (Chen et al., 2020). In this ecosystem, surface water infiltrates underground, and subsurface runoff tends to exceed surface runoff. Southwest China is the most concentrated area of karst landforms (Zhang et al., 2017). The cultivated land is thin and easily disturbed by the environment, such as soil erosion, overcultivation, and excessive application of chemical fertilizers and pesticides. This situation leads to low productivity of cultivated land in karst areas, and the use rate of nitrogen fertilizer is as low as 30% (Ju, 2014; Yang et al., 2021). The main reason is the migration and transformation of nitrogen fertilizer in the soil (Wang et al., 2023). After nitrogen enters the soil, in addition to being absorbed by crops, some nitrogen remains in the soil as inorganic and organic nitrogen, and the vast majority is lost through various pathways, including leaching, runoff, and gas. Studies have shown that applying nitrogen fertilizers to the soil in karst areas has a low retention rate and a high nitrogen leaching rate (Zhang et al., 2021). This condition leads to the deterioration of soil structure and fertility (Bai et al., 2020), resulting in air, water resources, and agricultural non-point source pollution problems (Savci, 2012; Chandini et al., 2019). Therefore, it is crucial to study the characteristics of nitrogen fertilizer migration and transformation, adopt effective fertilizer management methods, improve the nitrogen fertilizer use rate, and reduce nutrient loss.

Many scholars have studied the effects of different soil types, soil moisture, soil pH, fertilization methods, and fertilizer properties on soil nitrogen transfer and transformation (Wallace et al., 2019; Dromantienė et al., 2020). For example, NH_4_^+^-N produced by urea in paddy soil in an anaerobic soil layer can be protected from nitrification and denitrification. Urea application in deep soil can improve N absorption, and most NH_4_^+^-N remains in the rice root zone (Wallace et al., 2019). The 10–20 mm precipitation levels were simulated in the 5–20°C soil temperature range. The NO_3_^−^-N content in the 0–15 cm soil layer increased with soil temperature and rainfall when urea was applied. In contrast, the NH_4_^+^-N content decreased. An effective way to improve the nitrogen use rate of crops is by combining fertilizers and inhibitors, which could delay nitrification and reduce nitrogen loss (Wallace et al., 2019). Still, nitrification inhibitors and urease inhibitors are generally used. Nitrification inhibitors inhibit the bacterial activity of nitrite in soil, inhibit nitrification, and delay the transformation of NH_4_^+^-N to NO_3_^−^-N, thus reducing nitrogen loss (Kim et al., 2012). Urea can be hydrolyzed by urease after it is applied to the soil. Urease inhibitors slowed down the hydrolysis rate of urea and reduced the volatilization and nitration of ammonium nitrogen by competing with urease active sites (Cantarella et al., 2018). Liu et al (Liu  et al., 2022). investigated the nitrification mechanism of different nitrification inhibitor types in red soil, rice soil, and tidal soil. They found that nitrification inhibitors were able to inhibit the conversion of NH_4_^+^-N to NO_3_^−^-N in both rice and tidal soils and that the inhibition effect of tidal soil was better than that of rice soil. In contrast, the inhibition effect of nitrification on red soil was not obvious. Increasing the concentration of oxalic acid in the rhizosphere of the soil in karst areas can strengthen the ability of plants to obtain nutrients in the karst ecosystem. Oxalic acid significantly accelerates the mineralization and availability of nitrogen and significantly increases the concentration of total inorganic nitrogen (Yuan et al., 2018). Soil improvement with oxalic acid and inhibitors (DMPP and NBPT) can reduce nitrogen loss and improve the nitrogen use rate.

When nitrogen fertilizers are applied to soil, fertilizer microsites are formed near the fertilizer, with nutrient concentrations significantly different from the soil overall. In the fertilizer microsites of tidal soils, most of the applied phosphorus was immobilized, mainly within 2 mm of the soil at the point of application (Du et al., 2012). In the fertilizer microsites of Paddy soil, water-soluble phosphorus, and effective phosphorus gradually decreased with increasing temperature (Chen et al., 2014). Soil types are mainly yellow-brown, yellow, and red soils in karst areas (Luo et al., 2023). Yellow-brown soil is a critical agricultural area, producing various food and cash crops. However, a study on the migration and transformation of nitrogen fertilizer in infertile microsites of yellow-brown soil in karst areas is needed. Yellow-brown soil was used as the study object in this experiment. The effect of nitrogen fertilizer combined with soil improver and nitrogen fertilizer synergist on nitrogen migration and the transformation level of soil in karst areas was studied through an indoor simulated soil column culture for 30 days. The migration law of nitrogen in the soil was clarified, providing theoretical guidance and a scientific basis for efficiently utilizing nitrogen in agricultural production.

## 2 Materials and methods

### 2.1 Experimental soil

The soil sample was yellow-brown soil, which was collected from the Tian Yang District, Baise City, Guangxi Zhuang Autonomous Region (106°22’14”–107°8’32” E, 23°28’20”–24°6’55” N.), located in the middle of Youjiang Valley in western Guangxi, belonging to the low latitude south subtropical monsoon climate. The experimental soil was from karst areas in the southern rocky mountainous region, and surface soil 2–3 cm deep from the ground was collected by multi-point mixed sampling. The pH value of the mixed soil sample was 6.37, the organic matter content was 5.42 g/kg, the total nitrogen content was 0.56 g/kg, and the field water capacity was 33.3%. Oxalic acid, DMPP, NBPT, and four kinds of nitrogen fertilizers (analytically pure) were purchased from Tianjin Zhiyuan Chemical Reagents Co., Ltd.

### 2.2 Test method

#### 2.2.1 Experimental design

A soil sample bag with a height of 30 cm and an inner diameter of 10 cm was adopted. The soil required to fill a 21 cm soil column was calculated as 850 g, and the air-dried soil treated in the early stage was weighed and evenly filled to form a soil column. The soil was treated with water injection to ensure that the water content in the bag was 30%, 40%, and flooded, respectively, and stood for 24 h. Each soil water content was treated as follows: local farmers commonly use urea, ammonium bicarbonate, ammonium bicarbonate, and ammonium sulfate. Four nitrogen fertilizers (i.e., ammonium bicarbonate, ammonium sulfate, ammonium chloride, and urea) were treated with oxalic acid, DMPP, and NBPT, respectively (A1 ammonium bicarbonate, A2 ammonium bicarbonate + oxalic acid, A3 ammonium bicarbonate +DMPP, A4 ammonium bicarbonate +NBPT, collectively referred to as A treatment; B1 ammonium sulfate, B2 ammonium sulfate + oxalic acid, B3 ammonium sulfate +DMPP, B4 ammonium sulfate +NBPT, collectively referred to as B treatment; C1 ammonium chloride, C2 ammonium chloride + oxalic acid, C3 ammonium chloride +DMPP, C4 ammonium chloride +NBPT, collectively referred to as C treatment; D1 urea, D2 urea + oxalic acid, D3 urea +DMPP, D4 urea +NBPT, collectively referred to as D treatment). Each treatment was replicated three times. The mixture was applied to the surface of the soil column after resting. Then, the soil was covered at 135 g, the height was 3 cm, and the specific amount of water injected was calculated according to the quality of the covering soil. The mouth of the soil column was wrapped and sealed with a rope, and five small holes with a diameter of about 1 cm were pricked with needles to ensure ventilation, avoid an anaerobic environment, reduce water loss, and maintain soil water content during cultivation. Then, the soil column was placed horizontally indoors for 30 days.

#### 2.2.2 Sampling collection

**Figure 1:**
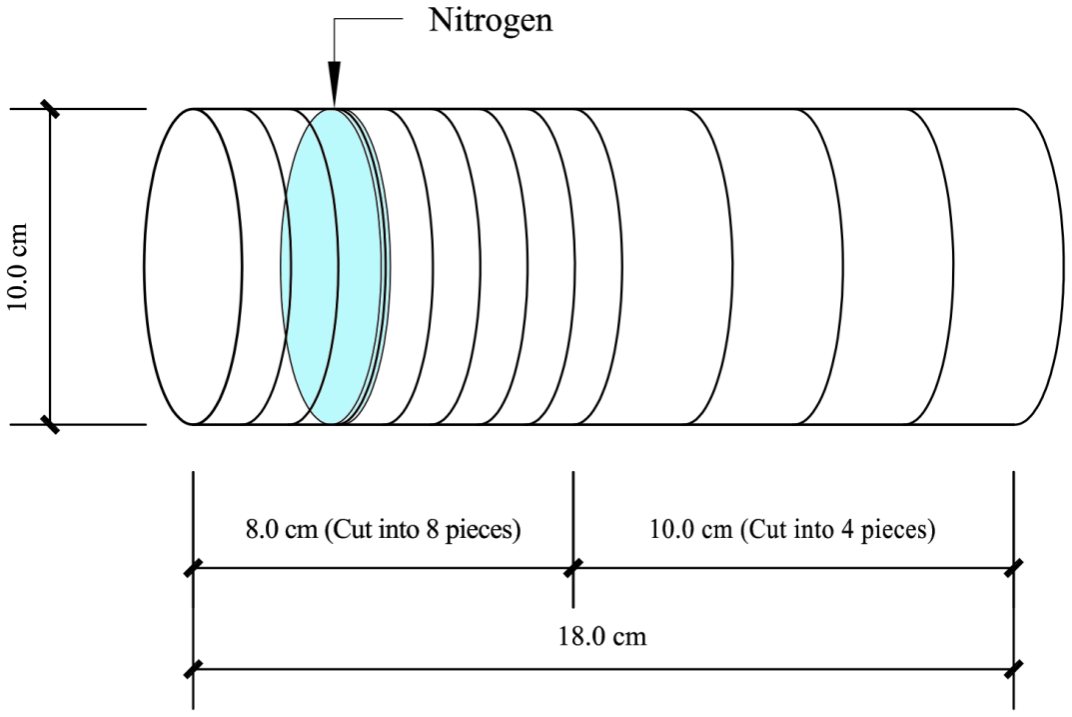
Horizontal placement and cutting diagram of soil column.

Samples were collected after the completion of the 30-day culture period. The soil column was placed horizontally. A 1 cm-wide soil layer was cut vertically at the binding mouth. Then, eight soil samples with a width of 1 cm were cut under the condition that the soil profile of each soil sample was consistent. Next, three soil samples with a width of 2.5 cm were cut successively from the cut-binding mouth to the bottom of the soil column. The remaining 3 cm of soil in the soil column bag was one part (**Figure 1**). The 12 cut soil samples were marked from left to right, −3, −2……10, 12.5, and 15 cm, respectively. Air-dried, ground, and sealed for testing after passing through a 20-mesh screen.

#### 2.2.3 Sample detection

The ammonium nitrogen (NH_4_^+^-N) determination was conducted using Nessler’s Reagent Spectrophotometry (Shang et al., 2009), with a 2mol/L KCl solution for extraction and colorimetric measurement at 420 nm. The determination of nitrate nitrogen (NO_3_^−^-N) was conducted by UV Spectrophotometry (Sempere et al., 2010), using 2mol/L KCl solution for extraction and measuring the absorbance at 220 and 275 nm wavelengths.

The ammonium nitrogen and nitrate nitrogen contents in the whole soil column were weighted by the thickness of each slice, and the ammonium nitrogen and nitrate nitrogen Cn(1, 2,. 12) contents in each soil column were summed (dN) to calculate the percentage rate.

Percentage rate (%) = dN/n × 1/1000, where N was the nitrogen content in various nitrogen fertilizers.

### 2.3 Data processing and analysis

All experiments were analyzed using a randomized complete block design, and ammonium nitrogen and nitrate nitrogen concentrations were analyzed using single-treatment factor designs. Excel 2019 and SPSS software were used for comparative processing and statistical analysis according to the obtained experimental data. The significance level was set as α = 0.05. Analysis and mapping were performed using Origin 2021.

## 3 Results

### 3.1 Soil NH_4_^+^-N and NO_3_^−^-N content under the application of ammonium bicarbonate combined with oxalic acid and inhibitors

**Figure 2:**
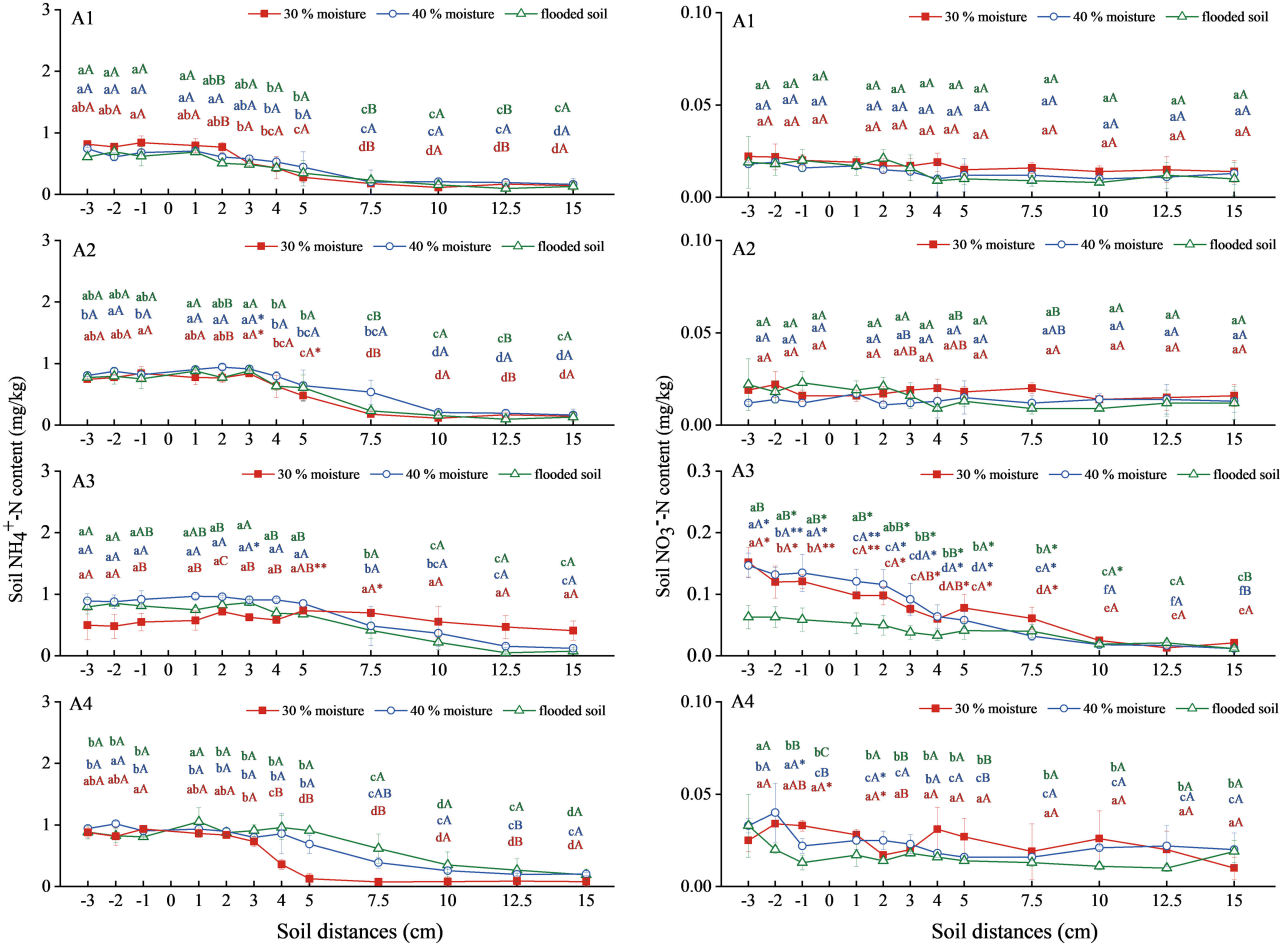
NH_4_^+^-N and NO_3_^−^-N content changes in soil under ammonium carbonate combined with oxalic acid and different inhibitors. N: A1 ammonium bicarbonate, A2 ammonium bicarbonate + oxalic acid, A3 ammonium bicarbonate +DMPP, A4 ammonium bicarbonate +NBPT. Different upper and lower case letters indicated highly significant (P < 0.01) and significant (P < 0.05) differences among treatments, respectively; Asterisks (*) and double asterisks (**) indicated significant (P < 0.05) and highly significant (P < 0.01) differences among treatments at the same moisture level.

Soil NH_4_^+^-N content of ammonium bicarbonate combined with oxalic acid and inhibitor had a flattening and decreasing trend (**Figure 2**). The NH_4_^+^-N of the three water-content soils in the ammonium bicarbonate treatment (A1) decreased at a soil-level distance of 2 cm. The decrease was delayed to a soil level distance of 3 cm in the ammonium bicarbonate treatment combined with oxalic acid treatment (A2). The ammonium bicarbonate combined with DMPP treatment (A3) extended the migration of NH_4_^+^-N in soil with 30% water content to a level distance of 7.5 cm, and the 40% water content and flooded state decreased after a level distance of 5 cm. Ammonium bicarbonate combined with NBPT treatment (A4) at 40% water content and flooded state, the migration distance continued to level 5 cm. Soil NH_4_^+^-N content increased slightly in the A2 and A3 treatments compared to the A1 treatment at 40% water content and flooded state, reaching a maximum of 0.943 mg/kg and 0.973 mg/kg at 40% water content. The A4 treatment showed an increase in soil NH_4_^+^-N content compared to A1 at 40% water content and flooded conditions, with a maximum of 1.021 mg/kg.

The overall soil NO_3_^−^-N content trend in the A1, A2, and A4 treatments was stable (**Figure 2**). A3 showed a trend toward a gradual decline in processing. There was no significant difference in soil NO_3_^−^-N content between the A1 and A2 treatments. The overall NO_3_^−^-N content of soil treated with A3 was 40% water content > 30% water content > waterlogging state, which was significantly higher than A1, and the content of level distance −3cm was the highest value, reaching 0.152, 0.147, and 0.063mg/kg, respectively. There was no significant difference between the A2 and A4 treatments and the A1 treatment.

Still, ammonium bicarbonate combined with oxalic acid or inhibitors could prolong the migration distance, and the combined inhibitors were able to improve the NH_4_^+^-N content of the soil. Ammonium bicarbonate combined with DMPP significantly increased soil NO_3_^−^-N content.

### 3.2 Soil NH_4_^+^-N and NO_3_^−^-N content under the application of ammonium sulfate combined with oxalic acid and inhibitors

**Figure 3:**
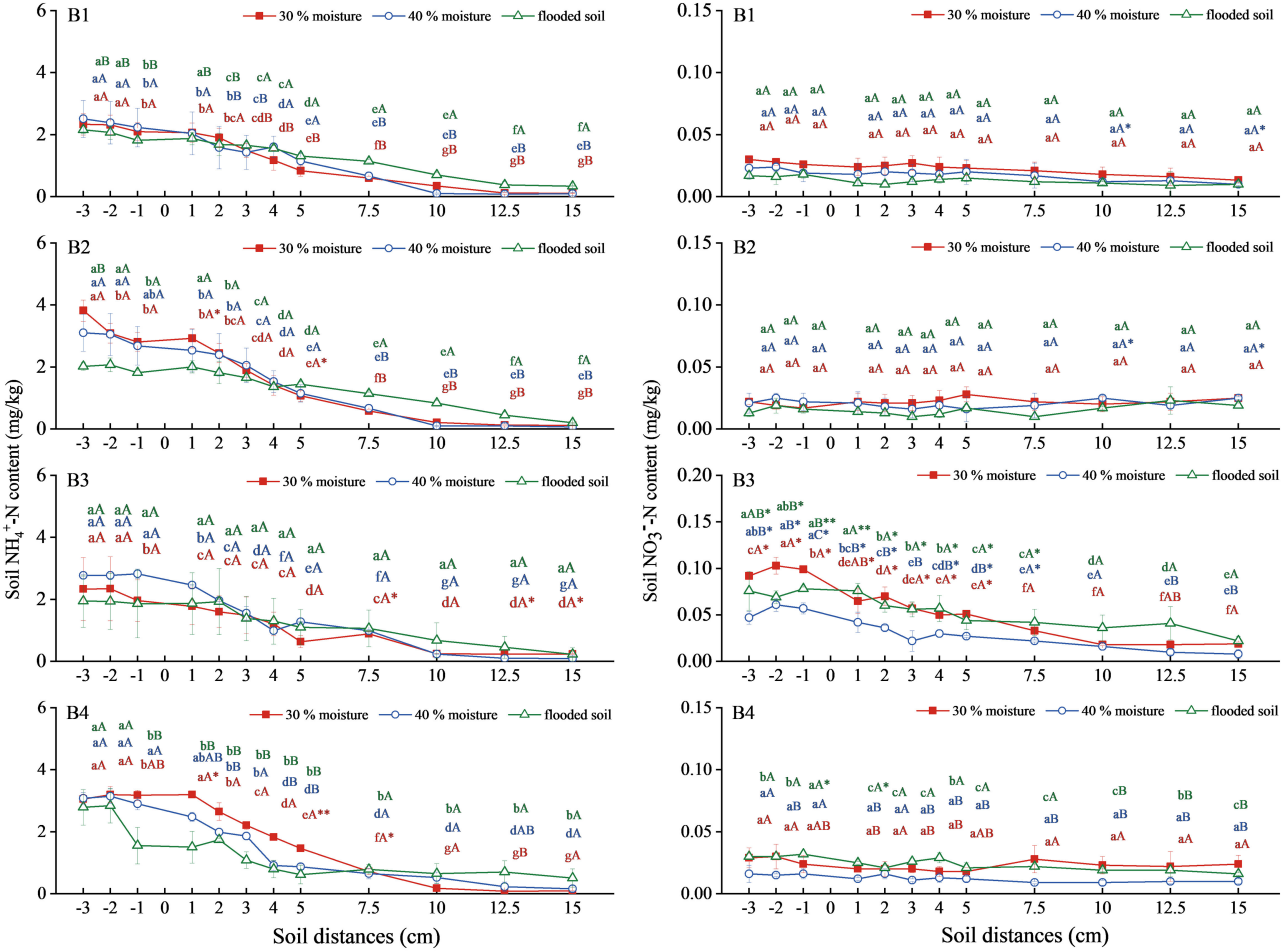
Changes of NH_4_^+^-N and NO_3_^−^-N content in soil with ammonium sulfate combined with oxalic acid and different inhibitors. N: B1 ammonium sulfate, B2 ammonium sulfate+oxalic acid, B3 ammonium sulfate+DMPP, B4 ammonium sulfate +NBPT. Different upper and lower case letters indicated highly significant (P < 0.01) and significant (P < 0.05) differences among treatments, respectively; Asterisks (*) and double asterisks (**) indicated significant (P < 0.05) and highly significant (P < 0.01) differences among treatments at the same moisture level.

The NH_4_^+^-N content in soil combined with ammonium sulfate with oxalic acid and inhibitor showed a steady downward trend (**Figure 3**). In ammonium sulfate treatment (B1), the soil NH_4_^+^-N contents were similar in all three soil types, regardless of water content. The NH_4_^+^-N content of soil treated with ammonium sulfate combined with oxalic acid at 30% and 40% water content of (B2) was higher than that of the flooded state. Still, the difference was insignificant and increased compared with the A1 treatment. The combinations of ammonium sulfate with DMPP (B3) at 40% water content and ammonium sulfate with NBPT (B4) at 30% water content were significantly higher than those of B1. The NH_4_^+^-N content in soil with 40% water content in B3 was the highest, 2.830mg/kg. The 30% water content was the highest in treatment B4, reaching 3.203 mg/kg.

The soil NO_3_^−^-N content treated with B1, B2, and B4 remained stable overall (**Figure 3**). Still, there was no significant difference in soil NO_3_^−^-N content among the three water contents. The B3 treatment showed a slow decreasing trend and significantly increased compared with the B1. The NO_3_^−^-N content in soil was 30% > flooded state > 40%, and 30% water content was the highest, 0.103mg/kg.

Overall, soil NH_4_^+^-N content increased under the 30% and 40% moisture treatments with oxalic acid and NBPT, respectively, compared to ammonium sulfate alone. At the same time, combining it with DMPP significantly increased the NO_3_^−^-N content in the soil.

### 3.3 Soil NH_4_^+^-N and NO_3_^−^-N content under the application of ammonium chloride with oxalic acid and inhibitors

**Figure 4:**
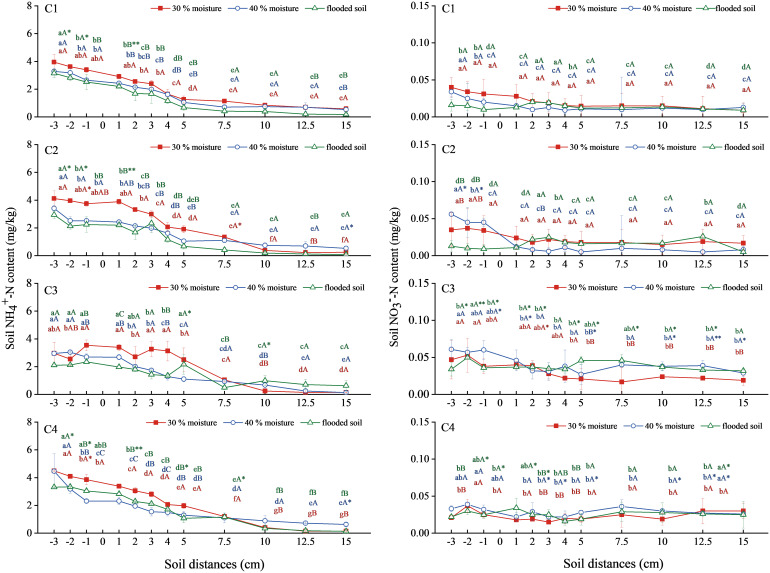
Changes in NH_4_^+^-N and NO_3_^−^-N content in soil with ammonium chloride combined with oxalic acid and different inhibitors. N: C1 ammonium chloride, C2 ammonium chloride + oxalic acid, C3 ammonium chloride +DMPP, C4 ammonium chloride +NBPT. Different upper and lower case letters indicated highly significant (P < 0.01) and significant (P < 0.05) differences among treatments, respectively; Asterisks (*) and double asterisks (**) indicated significant (P < 0.05) and highly significant (P < 0.01) differences among treatments at the same moisture level.

NH_4_^+^-N content in soil treated with ammonium chloride combined with oxalic acid and inhibitor showed a decreasing trend (**Figure 4**). Ammonium chloride (C1) and ammonium chloride combined with oxalic acid treatment (C2) decreased with increased water content. The NH_4_^+^-N content in soil with 30% water content was the most significant, reaching 3.953 mg/kg and 4.121 mg/kg at a level distance of −3 cm. The NH_4_^+^-N content in soil treated with ammonium chloride combined with DMPP (C3) was lower than that treated with C1. Treatment with ammonium chloride combined with NBPT (C4) was close to treatment C1, and the difference was insignificant.

The overall NO_3_^−^-N soil content in the C1, C2, C3, and C4 treatments was relatively gentle, with little change (**Figure 4**). There was no significant difference in soil NO_3_^–^N content between the C1 and C2 treatments. The C3 treatment increased significantly compared with the C1 treatment. Soil NO_3_^−^-N content at a level distance of −3 cm was the highest at 40% water content, reaching 0.061mg/kg. The NO_3_^−^-N content of soil treated with 40% water content and flooded condition in C4 slightly exceeded that in the treatment of C1 with the same water content but was not significantly different.

In summary, soil NH_4_^+^-N content increased when ammonium chloride was combined with oxalic acid at 30% water content. Adding nitrification inhibitors with ammonium chloride fertilization did not significantly increase the soil NH_4_^+^-N content. Still, it significantly increased the soil NO_3_^−^-N content, with DMPP being the most effective inhibitor.

### 3.4 Soil NH_4_^+^-N and NO_3_^−^-N content under the application of urea combined with oxalic acid and inhibitors

**Figure 5:**
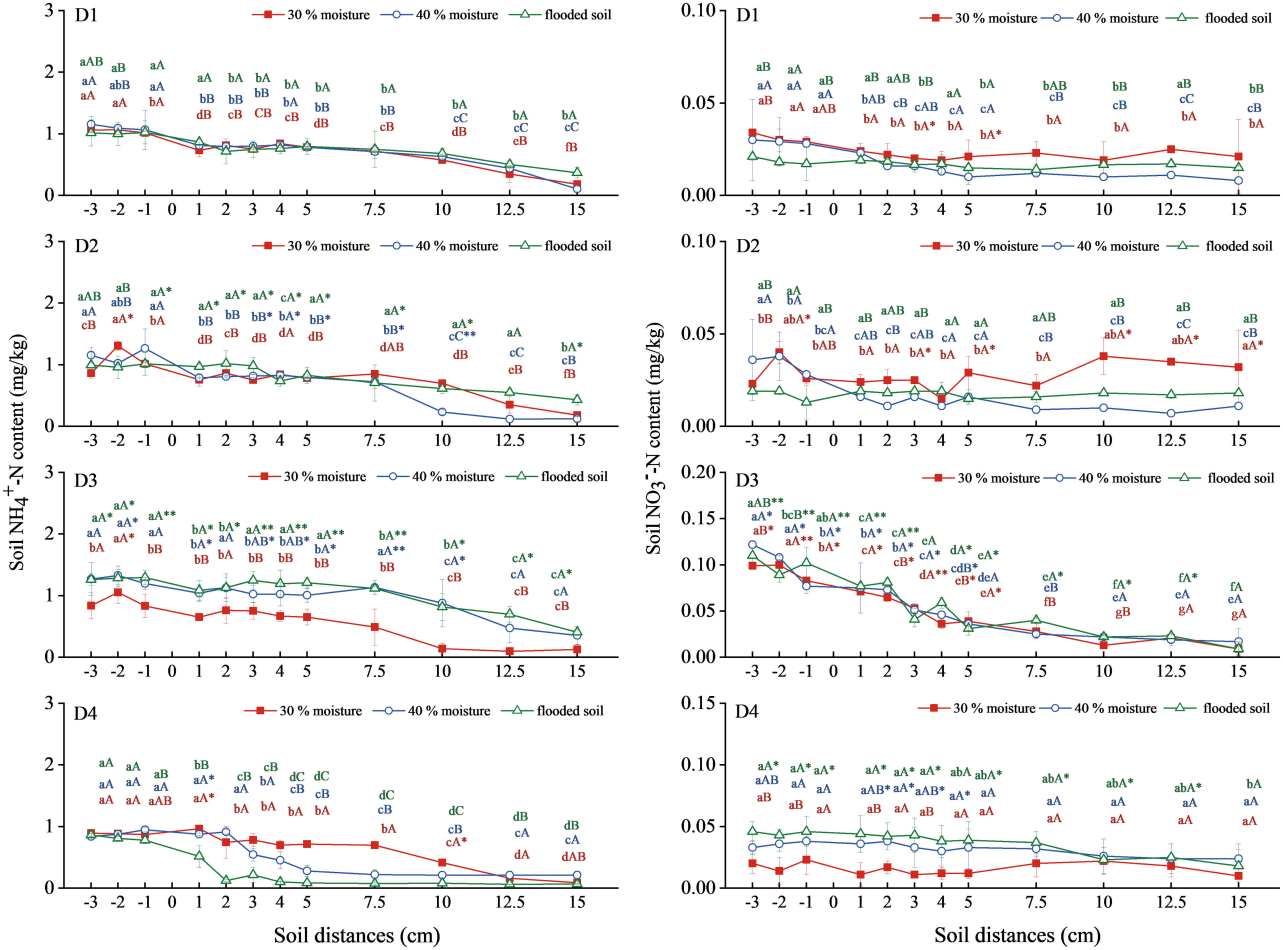
Changes in NH_4_^+^-N and NO_3_^−^-N content in soil with urea combined with oxalic acid and different inhibitors. N: D1 urea, D2 urea + oxalic acid, D3 urea +DMPP, D4 urea +NBPT. Different upper and lower case letters indicated highly significant (P < 0.01) and significant (P < 0.05) differences among treatments, respectively; Asterisks (*) and double asterisks (**) indicated significant (P < 0.05) and highly significant (P < 0.01) differences among treatments at the same moisture level.

The NH_4_^+^-N migration in the soil treated with urea, oxalic acid, and inhibitors gradually decreased (**Figure 5**). With urea alone (D1), urea combined with oxalic acid (D2), and urea combined with DMPP treatment (D3), soil NH_4_^+^-N decreased after migrating to a horizontal distance of 7.5 cm. At the same time, the migration distance decreased with the increase in water content in urea combined with NBPT treatment (D4). There was no significant difference in NH_4_^+^-N content among the three water contents in the D1 and D2 treatments. In the D2 treatment, the NH_4_^+^-N content was the highest in the soil with 30% water content, which was 1.962mg/kg at -2 cm horizontal distance. The soil NH_4_^+^-N content in the D3 treatment with 40% water content and submerged state was significantly higher than in D1. The highest values were 1.329 mg/kg and 1.295 mg/kg, respectively. There was no significant difference between urea combined with NBPT treatment (D4) and the other treatments.

The soil NO_3_^−^-N trend in treatments D1, D2, and D4 was gentle (**Figure 5**). In contrast, the soil NO_3_^−^-N trend in treatment D3 decreased steadily. There was no significant difference in soil NO_3_^−^-N among the three water content levels in the D1 treatment. In the D2 treatment, the soil NO_3_^−^-N content was most significant at 30% water content, with the highest at -2 cm horizontal distance, reaching 0.040mg/kg. Soil NO_3_^−^-N contents in the three water contents were similar, with the highest values being 0.100, 0.122, and 0.110mg/kg, respectively. Soil NO_3_^−^-N content in the D4 treatment increased with water content increase, and the flooding state was significantly higher than that of D1, up to 0.046mg/kg.

Therefore, urea combined with DMPP could significantly increase the NH_4_^+^-N content in the soil at 40% water content and submerged state. Urea combined with DMPP significantly increased the NO_3_^−^-N content in the soil, and the three water contents were close.

### 3.5 Percentage rates of soil NH_4_^+^-N and NO_3_^−^-N in nitrogen fertilizer under different treatments

**Figure 6:**
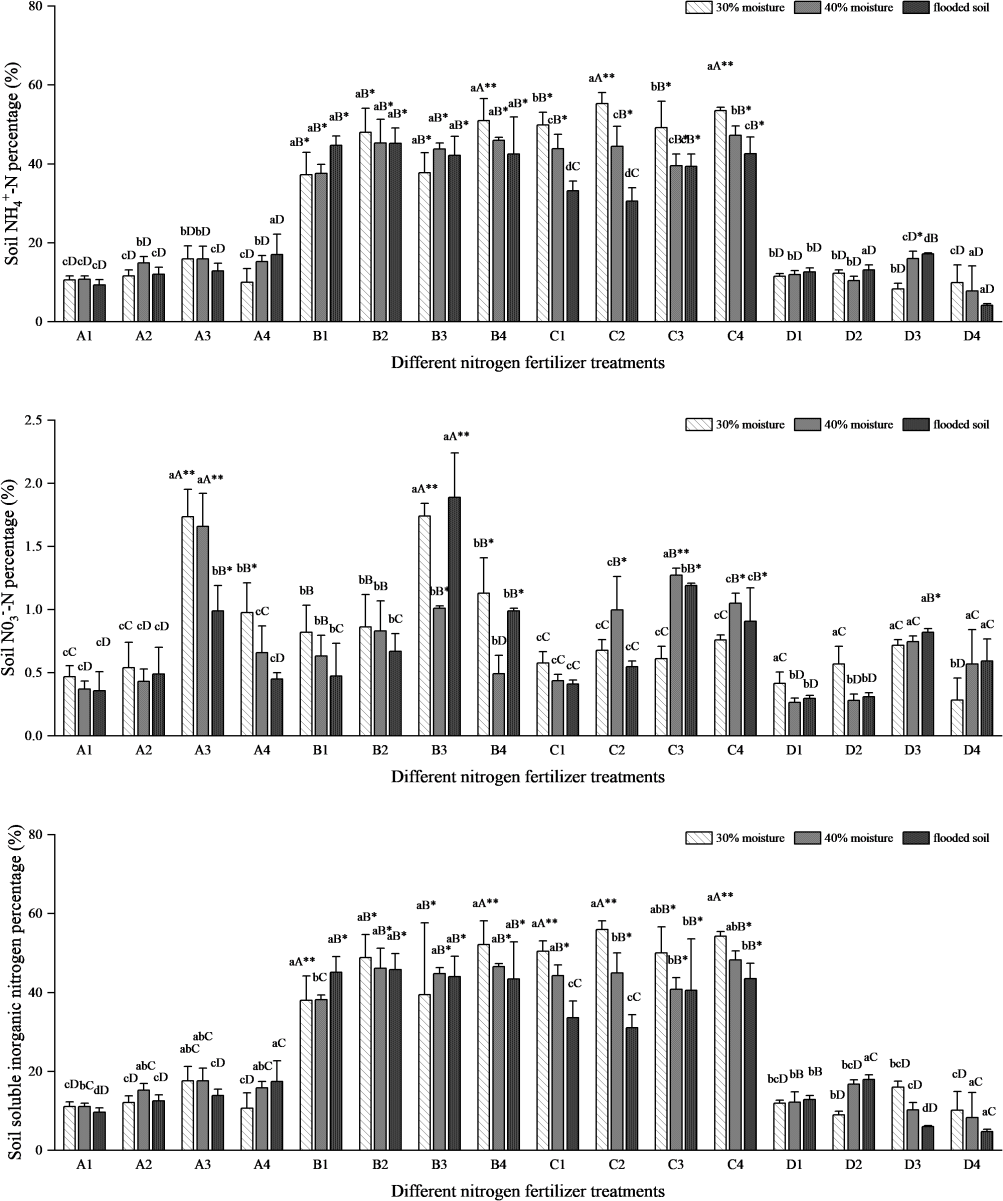
NH_4_^+^-N, NO_3_^−^-N, and soluble inorganic nitrogen percentage rates of different nitrogen fertilizer treatments. N: Lowercase letters indicate significant differences within groups, while uppercase letters indicate significant differences between groups of the same moisture level. Asterisks (*) and double asterisks (**) indicate significant and highly significant differences between treatments.

**Figure 6** shows that the soil NH_4_^+^-N percentage was less than 20% for treatments A and D and about 40% for treatments B and C. In the ammonium bicarbonate group, soil NH_4_^+^-N percentage decreased with increasing water content in A1 and A3 and increased and then decreased in A2. At the same time, A4 showed an increasing trend. In the ammonium sulfate group, the percentage of soil NH_4_^+^-N increased with increasing water content in B1, followed by a decreasing trend in B2 and B4, and an increase followed by a decrease in B3. The ammonium chloride group and the soil NH_4_^+^-N percentage in the C treatment group showed a decreasing trend with increasing water content. In the urea treatment group, soil NH_4_^+^-N percentage was not significantly changed in D1. Soil NH_4_^+^-N percentage first decreased and then increased with increasing water content in D2. This was followed by an increasing trend in the D3 treatment and a decreasing trend in D4. The soil NH_4_^+^-N percentage rate of the ammonium chloride group was the highest among the four nitrogen fertilizers, followed by the ammonium sulfate group, and the urea group was the lowest (**Figure 6**). After applying oxalic acid, the percentage of soil NH_4_^+^-N increased in the A2, B2, and C2 compared to the A1, B1, and C1. With DMPP, soil NH_4_^+^-N percentage increased in the A3 and D3 treatments compared to A1 and D1. With NBPT, soil NH_4_^+^-N percentage increased in some treatments of A4 and B4. In the A treatment, the NH_4_^+^-N percentage rate in the soil group under the flooded state of the A4 treatment was the highest, reaching 16.982%. In group B, the NH_4_^+^-N percentage rate in soil with 30% water content treated with B4 was the highest, reaching 50.99%, which increased by 13.77% compared with B1. In all C treatments, the NH_4_^+^-N percentage rate of 30% water content was the highest, and C2 and C4 were close, with 55.30% and 53.50%, respectively. In the D treatment group, the highest soil NH_4_^+^-N percentage rate was 17.16% in the flooded state of the D2 treatment.

The four nitrogen fertilizer treatments showed significant differences in soil NO_3_^−^-N percentage rates. However, the overall occupancy rates were all less than 2%. In 30% of B3 treatment and flooding, the percentage of soil NO_3_^−^-N was high, at 1.74% and 1.89%, respectively. Second, the 30% and 40% water content percentage rates in the A3 treatment were 1.74% and 1.66%, respectively. Moreover, the soil NO_3_^−^-N transformation rate in the other treatment groups was similar, and there was no obvious difference. The percentage rate of NO_3_^−^-N in the soil after four kinds of nitrogen fertilizers combined with DMPP was higher than that of oxalic acid and NBPT.

The soil-soluble inorganic nitrogen percentage fluctuation in all treatments was consistent with the trend of soil NH_4_^+^-N percentage. The percentage rate of soluble inorganic nitrogen in soil treated with B and C was the most significant among the four nitrogen treatments, at approximately 40%. The C2 and C4 treatments had the highest soil soluble inorganic nitrogen percentage under 30% moisture, which were 55.97% and 54.26%, respectively. Following the B4 treatment, the soil soluble inorganic nitrogen percentage was 52.12% at 30% moisture. The soil soluble inorganic nitrogen percentage in A3 combined with DMPP was significantly higher than in A1, and B4 combined with NBPT was higher than in B1. The soil-soluble inorganic nitrogen percentages were close between the C treatments. In the D treatment, the percentage of soil-soluble inorganic nitrogen was increased in D2 at 40% under flood conditions and in D3 at 30% water content compared to D1.

## 4 Discussion and conclusion

Many studies showed that urease inhibitors and nitrification inhibitors could improve the use efficiency of nitrogen fertilizer and reduce losses and had the potential to alleviate nitrogen pollution in agricultural production (Giovannini et al., 2009). The results showed that the NH_4_^+^-N and NO_3_^−^-N contents in the soil column treated with oxalic acid and inhibitors were different in different water contents. It was easy to produce a negative charge on the surface of the soil colloid, NH_4_^+^-N could be adsorbed by the soil colloid, and NO_3_^−^-N could not be adsorbed and easily lost with water. The migration sites of NH_4_^+^-N in A3 and A4 treatments were prolonged in ammonium bicarbonate treatment. This result indicated that ammonium bicarbonate combined with DMPP and NBPT could inhibit the activity of nitrifying bacteria and extend the fertilizer efficiency and migration distance of ammonium bicarbonate to some extent. There was no significant difference in soil NH_4_^+^-N content during treatment after adding ammonium sulfate with oxalic acid (DMPP). According to WANG and TORRALBO et al (Torralbo et al., 2017; Wang et al., 2018), ammonium sulfate application could maintain the acidity of the rhizosphere. At the same time, DMPP had a short half-life in acidic soils and was most effective in suppressing nitrification in viscous soils with high pH values (Cui et al., 2021). Hence, adding DMPP to ammonium sulfate did not result in any significant changes in soil NH_4_^+^-N content. The soil NH_4_^+^-N content with ammonium chloride and 30% oxalic acid was higher than that with other nitrogen fertilizers in the C treatment; with the increase in water content, NH_4_^+^-N content decreased, and there was no significant change after the addition of inhibitors. SOURI and VIEIRA MEGDA et al (Souri, 2010; Vieira Megda et al., 2014). found that in addition to nitrification inhibitors, chloride could also significantly inhibit nitrification by microorganisms in soil by increasing Cl^−^ content in the soil, and the inhibition was positively correlated with chloride concentration in soil. Therefore, the NH_4_^+^-N content of ammonium chloride and the inhibitor did not increase significantly in the C treatment. In the D treatment, the NH_4_^+^-N content of urea combined with DMPP increased at each point at 40% water content and flooded state. Although urea would rapidly hydrolyze to NH_4_^+^-N after application in wet soil, NH_4_^+^-N produced in the anaerobic soil layer was reduced by nitrification and denitrification due to anoxia (Siddique et al., 2019). Cantarella et al. (2018) found that NBPT showed high efficiency in delaying urea hydrolysis under anaerobic conditions. However, urea combined with NBPT had no significant effect on nitrification in the soil in this experiment. This result may be due to the influence of soil pH and organic carbon on soil urease and nitrification inhibitors in karst areas (Luchibia et al., 2020).

The differences in NH_4_^+^-N content of soil treated with four nitrogen fertilizers may be attributed to the different rates of NH_4_^+^-N provided for nitrification after hydrolysis of varying nitrogen fertilizers (Luchibia et al., 2020). Ammonium carbonate, ammonium sulfate, and ammonium chloride were all forms of nitrogen fertilizer containing ammonium ions (NH_4_^+^) or ammonia (NH_3_) (Yang et al., 2016). Ammonium carbonate was unstable and volatile, and it could evaporate even at room temperature. The nitrogen content of ammonium carbonate was lower than that of ammonium sulfate and ammonium chloride as it evaporates faster at high temperatures and with high moisture and better ventilation. As an amide nitrogen fertilizer, crops can only absorb and utilize urea after hydrolyzing into ammonium carbonate or ammonium bicarbonate (Wang, 2021). Therefore, its ammonium nitrogen content was lower than that of the first three fertilizers.

The NO_3_^−^-N content in the soil was the highest under the combined application of DMPP. The NO_3_^−^-N content of ammonium bicarbonate combined with oxalic acid and nitrification inhibitor was higher than that of other nitrogen treatments, and NO_3_^−^-N content decreased with the increase of water content. Under the treatment of ammonium sulfate, ammonium chloride, and urea combined with DMPP, the NO_3_^−^-N content in soil was higher than that of oxalic acid in each nitrogen fertilizer. The NO_3_^−^-N accumulation in the soil was high when ammonium bicarbonate and ammonium sulfate with DMPP were applied in this experiment. At the same time, Yan et al (Yu et al., 2020) studied NO_3_^−^-N content in soil. They found that the best fertilizer N forms of DMPP were ammonium sulfate and urea. This result may be because the effect of the nitrification inhibitor was affected by multiple factors, such as soil PH value and nitrogen application rate (Shi et al., 2016).

Ammonium bicarbonate combined with oxalic acid or inhibitors could prolong the horizontal migration distance of soil NH_4_^+^-N in karst areas and, combined with inhibitors, increase the NH_4_^+^-N content in the soil. Combining ammonium sulfate with oxalic acid or NBPT effectively increased the NH_4_^+^-N content in the soil at 30% and 40% water content. Urea combined with DMPP increased NH_4_^+^-N content in soil with increased water content. Ammonium chloride combined with inhibitor was not significant. Four nitrogen fertilizers combined with DMPP significantly increased the NO_3_^−^-N content in the soil. Ammonium chloride combined with oxalic acid can make the percentage rate of NH_4_^+^-N in soil reach 55.31%. The NO_3_^−^-N percentage rate in soil with ammonium bicarbonate combined with DMPP reached 1.89%. The percentage rate of soluble inorganic nitrogen in the soil was 55.97% when ammonium chloride combined with oxalic acid was applied with a 30% water content.

In conclusion, soil NH_4_^+^-N content was increased by ammonium bicarbonate combined with DMPP or NBPT, ammonium sulfate combined with oxalic acid or NBPT, and urea combined with DMPP. Four nitrogen fertilizers with DMPP increased NO_3_^−^-N content in the soil. Therefore, it effectively improved the percentage rate of nitrogen fertilizers and the effective use of nitrogen fertilizers. Furthermore, the experiment results will be truthed in the field experiment, which has been conducted. This result will be presented next paper.

## Data availability statement

The raw data supporting the conclusions of this article will be made available by the authors, without undue reservation.

## Author contributions

WJ: Formal analysis, Investigation, Software, Supervision, Writing – original draft. CQ: Formal analysis, Investigation, Software, Supervision, Writing – original draft, Conceptualization, Data curation, Funding acquisition, Methodology, Project administration, Resources, Validation, Visualization, Writing – review & editing.
